# Supplementary material for: Silene uniflora Extracts for Strawberry Postharvest Protection
Source: Plants (Basel). 2023 Apr 29;12(9):1846. doi: 10.3390/plants12091846 (PMC10180817; doi:10.3390/plants12091846)
Supplement: Supplementary file 1 [file plants-12-01846-s001.zip › plants-2331351-supplementary.docx]

*Silene uniflora* Extracts for Strawberry Postharvest Protection

L. Buzón-Durán, E. Sánchez-Hernández, P. Martín-Ramos, L.M. Navas-Gracia,
M.C. García-González, R. Oliveira, J. Martín-Gil

SUPPORTING INFORMATION

**Figure S1**. Infrared spectra of *S. uniflora* aerial plant organs.

**Figure S2.** GC−MS chromatogram of *Silene uniflora* extract.


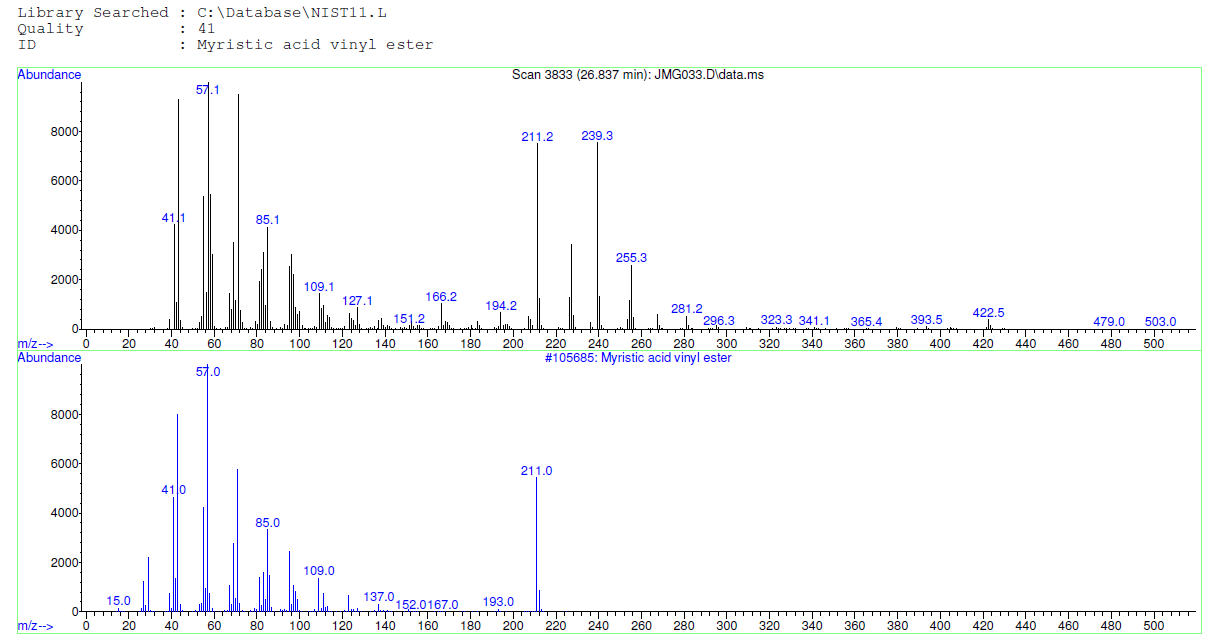


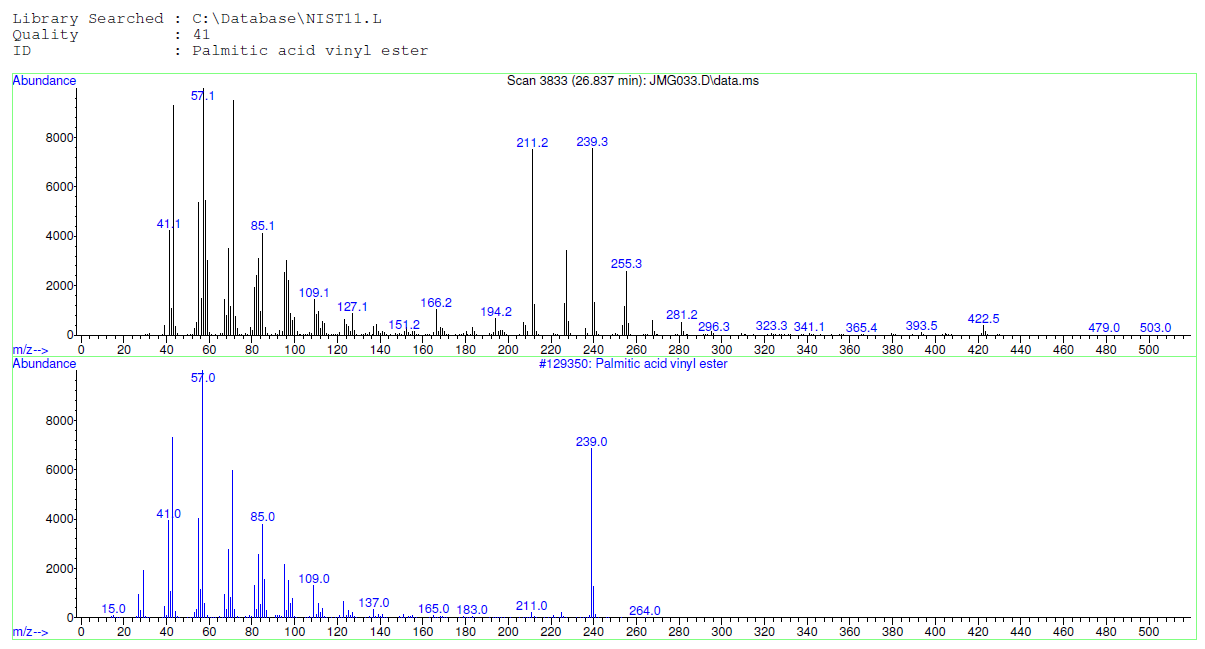


**Figure S3**. Comparison of MS spectra of two saturated fatty acid vinyl esters with that of the chemical species detected at RT=26.837 min.


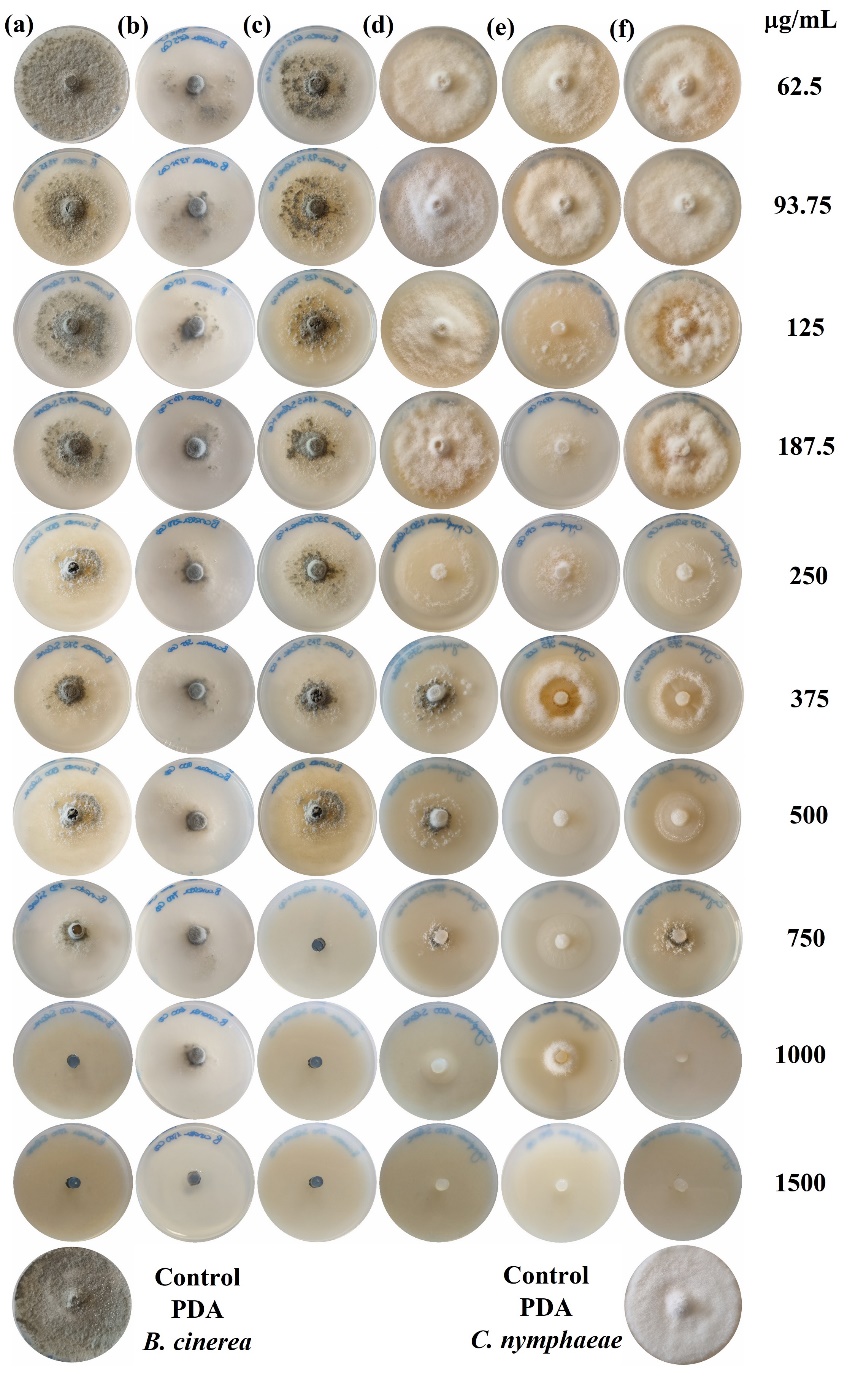


**Figure S4**. Radial growth of (**a−c**) *B. cinerea* and (**d−f**) *C. nymphaeae* in the presence of the three treatments, namely *S. uniflora* extract (**a, d**), chitosan oligomers (**b, e**), and COS−*S. uniflora* extract conjugate complex (**c, f**), at different concentrations (expressed in μg·mL^−1^).
